# Effectiveness and safety of oral anticoagulant therapy in a real-world cohort with atrial fibrillation: The SIESTA-A study protocol

**DOI:** 10.1371/journal.pone.0294822

**Published:** 2023-11-29

**Authors:** M. C. Montero-Balosa, J. A. Limón-Mora, A. Leal-Atienza, L. G. Luque-Romero, M. J. Aguado-Romeo, R. Isabel-Gómez, M. T. Molina-López

**Affiliations:** 1 Servicio de Farmacia de Atención Primaria, Distrito Aljarafe y Sevilla Norte, Servicio Andaluz de Salud, Sevilla, Spain; 2 Coordinación de Gestión y Evaluación, Servicio Andaluz de Salud, Sevilla, Spain; 3 Fundación Pública Andaluza para la Gestión de la Investigación en Salud de Sevilla (FISEVI), Consejería de Salud y Consumo, Sevilla, Spain; 4 Unidad de Investigación, Distrito Aljarafe y Sevilla Norte, Servicio Andaluz de Salud, Sevilla, Spain; 5 Centro de Transfusión de Tejidos y Células de Sevilla, Red Andaluza de Medicina Transfusional de Tejidos y Células, Consejería de Salud y Consumo, Sevilla, Spain; 6 Agencia de Evaluación de Tecnologías Sanitarias de Andalucía, Consejería de Salud y Consumo, Sevilla, Spain; 7 Servicio de Farmacia de Atención Primaria, Distrito Sevilla, Servicio Andaluz de Salud, Sevilla, Spain; Nanjing Drum Tower Hospital: Nanjing University Medical School Affiliated Nanjing Drum Tower Hospital, CHINA

## Abstract

**Introduction:**

Oral anticoagulants (OACs) are first-line drugs for stroke prevention in patients with atrial fibrillation (AF). The introduction of new lines of therapy with direct oral anticoagulants (DOACs) has led to a decreased use of vitamin K antagonists (VKAs). Comparative analyses of DOACs in clinical trials are scarce and the comparator has mostly been warfarin. Their impact on health outcomes in observational studies has not always been consistent. The aim of this study is to evaluate the effectiveness and safety of DOACs and VKAs in patients with AF using Real-World Data (RWD).

**Methods and analysis:**

Population-based retrospective cohort study using RWD from actual practice. Period: January 2012-December 2020. Inclusion criteria: patients with AF who had not taken OACs in the previous 12 months. Exclusion criteria: <40 years, with severe mitral stenosis, or valvular heart disease or aortic and/or mitral valve procedures. Data source: The Andalusian Population Health Database, Spain. Outcome measures: a) Effectiveness: ischaemic stroke, transient ischaemic attack, systemic and pulmonary embolism, and death; b) Safety: gastrointestinal and intracranial haemorrhaging; Independent variables: age, sex, comorbidities, medication and health resource use, CHA_2_DS_2_-VAS_C_, HAS-BLED, and analytical tests. Statistical analysis: crude incidence analysis, survival models, Kaplan-Meier, Cox regression analysis adjusted for possible confounding and paired analysis by propensity score matching.

## Introduction

Atrial fibrillation (AF) is the most common type of cardiac arrhythmia and the main cause of thromboembolic strokes and systemic embolism [1, 2]. Antithrombotic drugs are a cornerstone of the management of this disease. The treatments recommended by clinical practice guidelines for the prevention of strokes are oral anticoagulants (OAC), which include vitamin K antagonists (VKA) (warfarin and acenocoumarol) and the new direct anticoagulants (DOAC) (dabigatran, rivaroxaban, apixaban and edoxaban) [3, 4]. In recent years, however, DOAC prescriptions have largely replaced those of VKAs.

Clinical trials evaluating the efficacy and safety of DOACs have mostly used warfarin as a comparator drug [5–8]. A meta-analysis of clinical trials showed that DOACs reduce stroke, intracranial haemorrhage and mortality [9].

Nevertheless, different observational studies based on Real-World Data (RWD) have provided new evidence regarding the comparative safety and effectiveness of these drugs. Their findings are not always consistent. In a Danish cohort [10], Nielsen *et al*. found no significant differences in the effectiveness and safety profile of DOACs versus warfarin, except for dabigatran, which was associated with less bleeding. Lip *et al*. [11] showed, in patients with AF and high risk of gastrointestinal bleeding, that DOACs were associated with lower rates of vascular stroke and systemic embolism but had varying risks of major bleeding compared with warfarin. These results were similar to those obtained by Sheth *et al*. [12].

Other observational studies have compared the effectiveness and safety of different DOACs. Durand *et al*. [13] conducted a meta-analysis and found that patients on apixaban showed a lower risk of stroke and bleeding versus rivaroxaban, and similarly versus dabigatran. Ingason *et al*. [14] concluded that rivaroxaban was associated with an increased risk of gastrointestinal bleeding compared to dabigatran and apixaban. Acenocoumarol is the most widely used VKA in countries like Spain and Portugal, and has not been evaluated in clinical trials. Rodríguez-Bernal *et al*. have studied the effectiveness and safety profile of DOACs against acenocoumarol. They found no differences in clinical outcomes between DOACs and acenocoumarol overall, except in the case of intracranial haemorrhage, where the risk was lower with dabigatran and rivaroxaban than with acenocoumarol [15].

Acenocoumarol and warfarin have different half-lives and it is not known how this may affect outcomes in RWD studies. Dalmau *et al*. [16] found there was no association between poor anticoagulation control and the type of VKA treatment administered. Besides that, a new DOAC, edoxaban, came on to the market and it has not been included in RWD studies. The objective of the study is to evaluate the comparative effectiveness and safety of acenocoumarol, warfarin, dabigatran, rivaroxaban, apixaban and edoxaban in patients diagnosed with AF under routine clinical practice.

## Methods and analysis

### Study design

This is an observational retrospective cohort study of regionwide population of all patients with atrial fibrillation, who were newly prescribed an OAC: acenocoumarol (reference treatment), warfarin, dabigatran, rivaroxaban, apixaban, or edoxaban.

### Population and setting

The study will be carried out in the region of Andalusia, where the Public Health System of Andalusia) (PHS-A) provides health coverage to a population of 8.4 million inhabitants.

**Fig 1:**
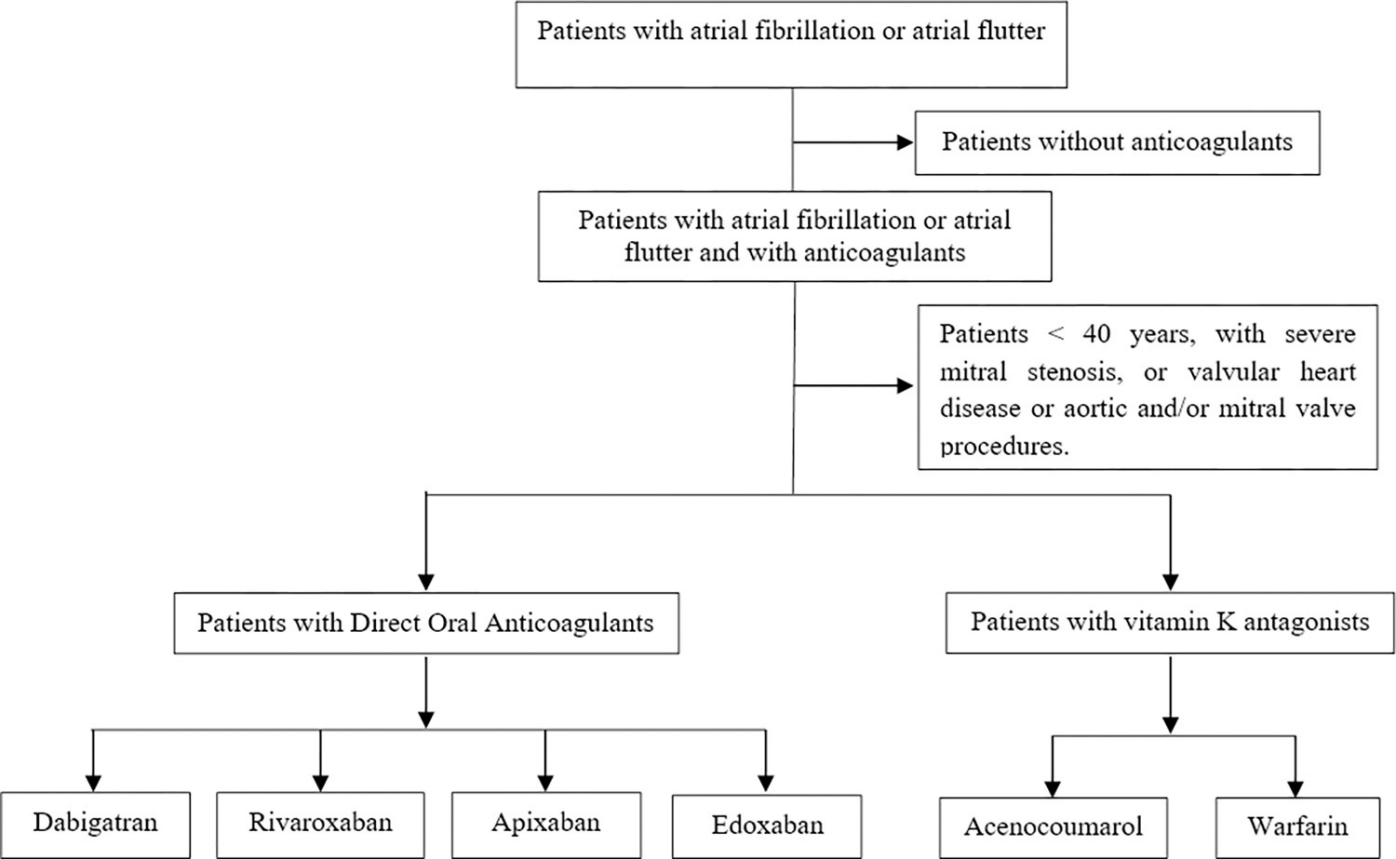
Patient selection criteria.

The patients in the study will be those seen in consultations at any level of health care of the PHS-A (primary care and hospital) between January 2012 and December 2020 (Fig 1) who meet the following inclusion/exclusion criteria:

Inclusion criteria (S1 Table):

- Diagnosis of AF or atrial flutter.
- Individuals initiating treatment with OACs under routine clinical practice conditions (*naive* population). Only new users of OAC (incident cases) will be included, even if they have taken OAC in the past, provided they stopped taking it during the 12 months prior to inclusion in the study. Patient data from 2012 onwards will be used.

Exclusion criteria (S1 Table):

- Under 40 years of age
- Patients with severe mitral stenosis
- Patients with valvular heart disease
- Patients undergoing aortic and/or mitral valve procedures.

This is a pathology that tends to present, in the majority of cases, after the age of 40 years [5–8, 15]. By selecting patients > 40 years, we consider that the conclusions for the majority adult population, which is the target population of our study, would gain in homogeneity.

### Data sources

**Fig 2:**
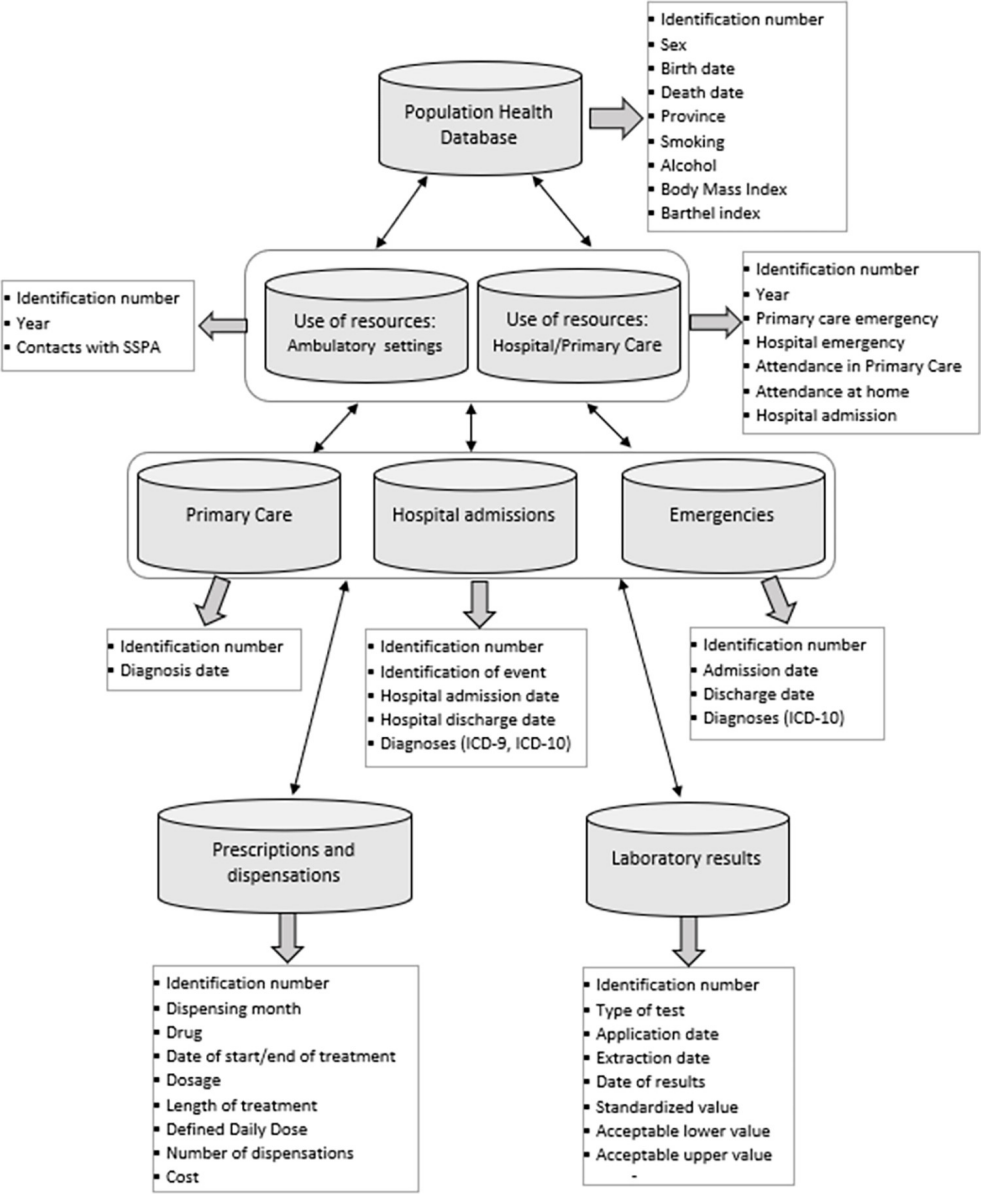
Data sources and linkage between databases. PHS-A: Public Health System of Andalusia; ICD: International Classification of Diseases. User database contains the patient’s affiliation and socio-demographic data. Database on resource use and/or admissions for outpatient consultations, primary care, hospital care and emergencies. Database containing prescription and pharmacy dispensing data in primary and hospital care. Clinical tests database.

The data source will be the Andalusian Population Health Database (APHD) [17]. This is a population-based health information system that collects clinical data and data on the use of health resources for each individual receiving health care in the Andalusian Health Service. This information is held in different databases that are linked by the unique health history number of each patient in the PHS-A (Fig 2):

The information will be extracted from the databases and transferred to a single file for analysis by the Excel, R and Stata statistical programmes. Patients will be anonymised for evaluation by the study investigators.

### Outcome measures

The pre-specified effectiveness outcomes will be the incidence of new diagnosis among the following list: transient ischaemic attack, systemic embolism, pulmonary embolism, ischaemic stroke and all-cause mortality, as a combined effectiveness endpoint.

Safety outcomes will be the incidence of new diagnosis of major haemorrhages leading to hospital admission (gastrointestinal and intracranial haemorrhages). They will be considered as a combined safety endpoint. Post-traumatic bleeding will be excluded.

All effectiveness and safety events will be associated with their dates of onset and identified using the corresponding International Classification of Diseases (ICD-9 or ICD-10 codes) (S2 Table).

Time-to-event will be defined as the number of days elapsed from the date of initiation of treatment (dispensing at pharmacy) until the date of the event, death or the end of study period (December/2020).

The following independent variables will be analysed according to relevance and availability in PHS-A databases:

- Type of OACs: date of start and end of treatment and dosages of the OACs under study (DOACs and VKA) will be collected.
- Sociodemographic data: age (time between date of birth and the event), Health Management Area, reference hospital, province.
- Contacts with the PHS-A (episode count and attendance indicators recorded in the last year and the annual average recorded during the study period) in primary care centres, hospitals, at home, and in the emergency department.
- Comorbidities: diagnosed health problems and their associated dates will be included (S3 Table). Comorbidity will be defined as the presence of an active diagnosis in the available databases (emergency, hospital and primary care) in the 12 months prior to the date of the event.
- Variables related to medication use: dates of start and end of treatment, dosage. The use of medication related to increased risk of bleeding or prevention of bleeding will be analysed according to the Anatomical Therapeutic Chemical (ATC) classification of the WHO Collaborating Centre for Drug Statistics Methodology (www.whocc.no/) (S4 Table).
- CHADS_2_ and CHA_2_DS_2_-VAS_C_ (Thromboembolic Risk Assessment) and HAS-BLED (Bleeding Risk Assessment) scales: the scores on these scales will be calculated at the date of the patient’s inclusion in the study, using the information available in the previous 12 months.
- All variables or diseases included in the CHADS2 and CHA2DS2-VASC scores will be considered for calculation of the total thromboembolic risk score. Diagnoses of “previous stroke”, “ischaemic stroke” (excluding haemorrhagic stroke), “TIA”, "systemic embolism", "pulmonary embolism" (hospital care diagnoses) and “thromboembolic events” (primary care diagnoses) will be grouped into a single section of the CHA2DS2-VASC scores. A history of “myocardial infarction", "angina pectoris" and "peripheral arterial disease" (diagnoses recorded in both hospital and primary care) will be considered as "vascular disease". The total score of the scale will be between 0 and 9 points.
- Intracranial haemorrhages and haemorrhagic strokes will be counted as "previous cerebrovascular accident" on the HAS-BLED Scale. Records with an ICD-9 or ICD-10 identification for renal or liver failure, or their primary care equivalents, will be collected. Safety outcome events of the study (intracranial or gastrointestinal haemorrhage) previously experienced by the patient will be considered as “Previous history of or predisposition to bleeding". "Labile INR" will not be included in calculations on this scale, since it applies to just one of the groups of medicines under study (VKA). Drugs predisposing to bleeding will be included in the calculation of bleeding risk (S4 Table). The “alcohol consumption” variable will not be included in the scale, as this information is not available in the vast majority of cases. The total score of the scale will be between 0 and 7 points
- Predictor variables will be analysed for associations with events based on information available in the previous 12 months.

### Data analysis plan

Cohorts will be compared by matching patients based on their propensity scores (PS) to be treated with acenocoumarol versus each of the four DOACs and versus warfarin in order to counteract the risk of selection bias when comparing the 5 different cohorts of OACs against the reference treatment cohort (acenocoumarol). This can be viewed as an attempt to emulate a randomized experiment (quasi-experimental study).

We consider that *propensity score matching* is of relevance in new RWD studies, and is more appropriate than other methods, such as *inverse probability of treatment weighting* (IPTW). After consulting the literature, matching cases by propensity score at the beginning of the study seems to be a more suitable epidemiological approach than first calculating a propensity score and assigning a weight to each case, before calculating the IPTW, unlike other publications on RWD in oral anticoagulation [10, 14, 15]. This strategy will help to control for selection bias by creating case-matched treatment groups that will be more comparable based on baseline patient characteristics.

A longitudinal registry will be created in a database that will later be imported with the Excel, R and Stata statistical packages together with the definitions of the variables described. The risk of events (effectiveness and safety) in patients treated with VKA and DOACs will be assessed.

Quantitative variables will be summarised as means and standard deviations, and in case of highly skewed distributions, as median and interquartile ranges (p25, p75). Frequency tables and percentages will be used for non-quantitative variables. Point estimates and 95% confidence intervals will be obtained for the different statistical analyses.

Contingency tables will be developed and the chi-square test, chi-square with continuity correction, and Fisher’s exact test (for 2x2 tables with small samples) will be applied to assess the relationship between two qualitative variables. In our analysis, parametric statistical tests will be used when variables fulfil the assumptions of normality and independence. Parametric tests are usually more powerful and sensitive when the data follow a normal distribution, allowing us to obtain more accurate and meaningful results. Non-parametric tests will be applied if the assumption of normality is not met.

Univariate survival analysis (for time-censored data) will be performed using Kaplan-Meier curves to represent the frequency of occurrence of different events or diseases over time and, where appropriate, in relation to different categories of a variable (age groups, sex, province, etc.) [18].

Survival probabilities, median and quartiles will be calculated, generating statistics and plotting survival functions for each study group.

The log-rank test will be used to test the equality of survival time distributions between different groups.

A preliminary univariate analysis will be performed to identify variables related to time-to-event occurrence (comparing event incidence density to person time to follow-up for different categories of variables).

To analyze this database, confounding variables identified in previous studies will be taken into account. Statistical analyses will include bivariate analysis (association between type of anticoagulant used and each of the other study variables); the Mantel-Haenszel test (analysis stratified by a third variable), as well as multivariate analysis.

A multivariate survival analysis will be performed using Cox proportional hazards or time-dependent Cox regression to control for the effect of confounding variables in the study. Cox regression will be used to create time-to-event models, considering both categorical and continuous predictor variables (covariates) as hypothetical predictors. Potential confounding variables include: age, sex, contacts with health service, comorbidities, concomitant medications, CHA2DS2-VASC and HAS-BLED scores, and previous ischemic and bleeding events.

Determination of the risk of outcomes among DOACs or warfarin versus acenocoumarol (reference) users will be obtained by calculating the specific instantaneous risk ratios for each comparison (Hazard Ratio) (HR) using multivariate Cox regression models. Adjusted survival curves will be used to represent how event risks will evolve over time after adjusting for different confounding variables.

A significance level of 0.15 will be used in preliminary univariate analysis to enable the selection of variables for inclusion in multivariate analysis. Once these variables have been selected, a multivariate analysis will be performed to select the final model. The analysis will be performed for both effectiveness and safety events. HRs and 95% confidence intervals (95% CIs) will be calculated for the selected variables once the application requirements have been validated.

The characteristics of the total study population and of each of the cohorts based on the first OAC used will be reported in the corresponding tables as percentages, means (standard deviation) or medians (interquartile range). Incidence rates will be calculated as the number of events divided by the corresponding denominator of person-time in follow-up.

**Fig 3:**
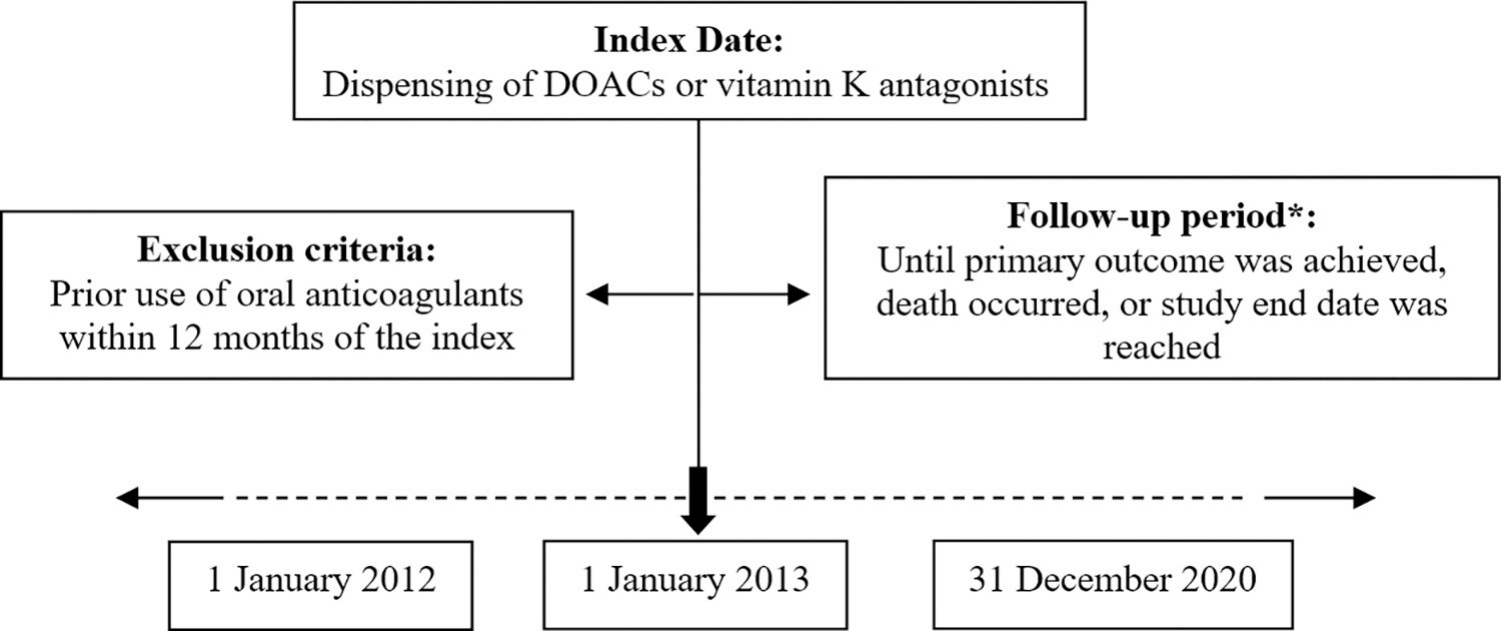
Cohort creation diagram. DOAC: direct oral anticoagulant. *Intention to treat analysis.

Intention-to-treat analysis will be carried out by categorising each patient according to the first anticoagulant treatment they start taking in the study period, provided that they have not taken any other anticoagulant for at least 12 months previously (“washout period”) and regardless of any possible changes in type of anticoagulant or non-adherence that may occur in the follow-up period (Fig 3).

To counteract the risk of selection bias when comparing the benefits of different treatments against the reference treatment (acenocoumarol), cohorts will be compared by matching individuals or patients based on their propensity scores (PS) for acenocoumarol versus each of the four DOACs and versus warfarin.

Measurement of medication effect or outcome for both effectiveness and safety variables will take into account the first recorded event (effectiveness or safety) among those included in the study.

The reporting of this study will abide by the Strengthening the Reporting of Observational Studies in Epidemiology (STROBE) statement for cohort studies [19].

### Registration details

The study protocol was submitted to the Spanish Agency of Drugs and Health Products (SADHP, the competent authority) and classified as an “Observational study” (SADHP number: 0004-2022-OBS; 12 January 2022).

## Ethics and dissemination

The study was approved by the Andalusian Biomedical Research Ethics Coordinating Committee (ABRECC), which considered that it complied with the ethical principles established for this type of study as set out in the Declaration of Helsinki (26 May 2020).

The results will be presented at national and international conferences and published in high impact journals.

### Project Data Protection Impact Assessment (DPIA)

A DPIA was required to obtain authorisation of the study by the ABRECC. This tool assesses the potential risks to which the processing of personal data may be exposed during the conduct of the study, and the provision of possible interventions or safeguards by the research team to minimise these risks. This assessment includes a description of the data lifecycle, an analysis of the necessity and proportionality of the processing, identification of threats and an assessment of inherent risk.

The research team included the following undertakings in the DPIA:

1. To use a dedicated computer system for data processing. Data will be stored in folders with restricted access to researchers on the corporate servers of the Regional Government of Andalusia. At no time will it be classified or stored in the cloud. No personal data will leave the corporate network of Regional Government of Andalusia.
2. To use the data only for research financed with public funds and carried out by a team of researchers from public bodies belonging to the Andalusian Health System.
3. To maintain patient confidentiality at all times, with researchers and those responsible for statistical analysis handling anonymised data at all times.
4. It will not be considered necessary or feasible to request informed consent from the patient due to the large volume of data to be handled.
5. The transfer of data between PHS-A and research team will be carried out by means of file transfer protocols between health ring servers belonging to the corporate network of the Regional Government of Andalusia. The research project team does not anticipate assignment or transfer of data to third parties.
6. There is a formal commitment not to seek to identify patients from encrypted or anonymised files.
7. All folders and files containing study data in the computer systems will be destroyed once the study has ended.
8. The Principal Investigator undertakes not to transfer data to third parties during the processing of the same.

## Strengths and limitations

### Limitations

The main limitation of the study is the information source itself, since the health records may be incomplete and underreporting may occur due to any of the following: random or accidental errors that are impossible to control for; systematic errors deriving from faulty measuring instruments, the observer, and so on; errors due to the patient being unable to remember all symptoms; errors due to incorrect descriptions of symptoms or diagnoses; errors due to professionals making a diagnosis without having all the information, or a lack of resources, or because the same resources are not available at all centres; classification or coding errors (of events or diagnoses); errors due to confusion (age, etc.); errors due to diagnostic tests giving false positives or false negatives and errors due to imprecise information due to non-quantifiable data (pain, improvement, etc.).

Secondly, this is a retrospective observational study based on real data from clinical practice, so that selection biases may be expected, more specifically, in the selection of OAC treatment, given that DOACs are subject to authorisation prior to dispensing, and dosage depends in turn on renal function, with different adjustments depending on the DOAC. Likewise, there may be differences in the use of acenocoumarol (vs warfarin) depending on the hospital reference area of the patient. This issue will be addressed by a cohort analysis matched by propensity score for treatment.

Thirdly, dabigatran was the first DOAC to be marketed and apixaban and edoxaban the last ones. We will address this problem by matching patients based on their PS and defining the follow-up period once these drugs were marketed.

There are other limitations. It is assumed that the medication dispensed is the medication that is taken by the patient, as it has been considered by other investigators [10, 13, 14, 16] but it cannot be rule out residual confounding. There may however be cases in which the patient obtains medication without a prescription or in a different region from the one in which the study is being conducted (Andalusia). It is not possible to record other medications with a bleeding risk that the patient may be taking that do not require a prescription (such as Aspirin®). However, the use of acetylsalicylic acid will be analysed, and other use of this drug is not expected to have a direct impact on the analysis. Socio-economic status will not be assessed since this data is barely reflected in the clinical records of patients. However, given that DOACs are subject to approval for the indication of non-valvular AF and that this is a universal public health service, socioeconomic status is not expected to affect the selection of type of anticoagulant. Finally, the analysis will be conducted in a particular region in southern Spain and may not extrapolate to other populations.

### Strengths

The PHS-A serves the vast majority of the Andalusian population, so that coverage of the system can be considered to be population-based. The study will make it possible to analyse a large sample of patients under real rather than ideal conditions (clinical trials), thereby allowing for extrapolation to the general population (high external validity).

To our knowledge, this will be one of the first studies to incorporate RWD to evaluate the effectiveness and safety of medicines in Andalusia. The methodology and knowledge gained can be extrapolated to studies involving other therapeutic groups and researchers.

The study using RWD will make it possible to establish clinically relevant associations, to identify adverse reactions to OACs at an early stage and to consider patient stratification to improve treatment response.

We highlight the long follow-up time for the analysis of events associated with the effectiveness and safety of OACs, as well as the absence of conflicts of interest, as there is no funding from the pharmaceutical industry.

The study researchers constitute a multidisciplinary group, which will make it possible to carry out an exhaustive analysis of the results and to draw conclusions from different healthcare perspectives, to ensure better implementation of the recommendations in routine clinical practice.

## Supporting information

S1 Table List of ICD-9 and ICD-10 codes to define inclusion and exclusion criteria. ICD: International Classification of Diseases.
(DOCX)

S2 Table List of ICD-9 and ICD-10 codes to define the primary outcome measures of effectiveness and safety. (DOCX)

S3 Table Definitions on comorbidity according to ICD-9 and ICD-10. AF: Atrial fibrillation; COPD: Chronic Obstructive Pulmonary Disease.
(DOCX)

S4 Table Concomitant medication according to Anatomical Therapeutic Chemical (ATC) codes. (DOCX)
