# Supplementary material for: Effectiveness and safety of oral anticoagulant therapy in a real-world cohort with atrial fibrillation: The SIESTA-A study protocol
Source: PLoS One. 2023 Nov 29;18(11):e0294822. doi: 10.1371/journal.pone.0294822 (PMC10686507; doi:10.1371/journal.pone.0294822)
Supplement: S1 Table — ICD: International Classification of Diseases. (DOCX) [file pone.0294822.s001.docx]

|  | **Diagnosis** | **Diagnosis Code** | | **Equivalence in Primary Care** |
| --- | --- | --- | --- | --- |
|  |  | **ICD-9** | **ICD-10** |  |
| **INCLUSION CRITERIA** | Atrial fibrillation | 427.31 | I48.0, I48.1, I48.2, I48.91 | FA |
|  | Atrial flutter | 427.32 | I48.3, I48.4, I48.92 | FLUTER |
| **EXCLUSION CRITERIA** | Severe mitral stenosis | 394.0, 394.2, 396.0, 396.1, 396.8 | I05.0, I05.2, I08.0, I08.1, I08.3, I34.2 | EST_MITRAL |
|  | Valvular heart disease or aortic and/or mitral valve procedures | V43.3  35.22, 35.24 (procedures) | Z95.2  02RF0JZ, 02RG0JZ (procedures) | PROTESIS_AOM |

**S1 Table. List of ICD-9 and ICD-10 codes to define inclusion and exclusion criteria**.

ICD: International Classification of Diseases.
