# Supplementary material for: Effectiveness and safety of oral anticoagulant therapy in a real-world cohort with atrial fibrillation: The SIESTA-A study protocol
Source: PLoS One. 2023 Nov 29;18(11):e0294822. doi: 10.1371/journal.pone.0294822 (PMC10686507; doi:10.1371/journal.pone.0294822)
Supplement: S2 Table — (DOCX) [file pone.0294822.s002.docx]

|  | **Diagnosis** | **Diagnosis Codes** | | **Equivalence in Primary Care** |
| --- | --- | --- | --- | --- |
|  |  | **ICD-9** | **ICD-10** |  |
| **Effectiveness Outcomes** | Transient ischaemic attack | 435.x | G45.x | AIT |
|  | Systemic embolism | 444.x, 445.x | I74.x, I75.x | --- |
|  | Pulmonary embolism | 415.1x, | I26.x | --- |
|  | Ischaemic stroke | 433.x1, 434.x1 | I63.x | --- |
| **Safety Outcomes** | Gastrointestinal haemorrhages | 456.0, 456.20, 531.0x, 531.2x, 531.4x, 531.6x, 532.0x, 532.2x, 532.4x, 532.6x, 533.0x, 533.2x, 533.4x, 533.6x, 534.0x, 534.2x, 534.4x, 534.6x, 535.x1, 537.83, 562.x2, 562.x3, 568.81, 569.3, 569.85, 578.x | I85.01, I85.11, K25.0, K25.2, K25.4, K25.6, K28.0, K28.2, K28.4, K28.6, K29.x1, K31.811, K55.21, K57.x1, K57.x3, K62.5, K66.1, K92.0, K92.1, K92.2 | HEM_DIGESTIVA |
|  | Intracranial haemorrhages | 430, 431, 432.x | I60.x, I61.x, I62.x | ICTUS_HEM |
|  |  | Cases excluded if prior traumatic brain injury (800-804, 850-854) | S02.1, S02.2, S02.3, S02.4, S02.6, S02.8, S02.9, S06.X |  |

**S2 Table. List of ICD-9 and ICD-10 codes to define the primary outcome measures of Effectiveness and Safety.**
