# Supplementary material for: Effectiveness and safety of oral anticoagulant therapy in a real-world cohort with atrial fibrillation: The SIESTA-A study protocol
Source: PLoS One. 2023 Nov 29;18(11):e0294822. doi: 10.1371/journal.pone.0294822 (PMC10686507; doi:10.1371/journal.pone.0294822)
Supplement: S3 Table — AF: Atrial fibrillation; COPD: Chronic Obstructive Pulmonary Disease. (DOCX) [file pone.0294822.s003.docx]

**S3 Table. Definitions on comorbidity according to ICD-9 and ICD-10.**

AF: Atrial fibrillation; COPD: Chronic Obstructive Pulmonary Disease.

|  | **Diagnosis** | **Diagnosis Codes** | | **Equivalence in Primary Care** |
| --- | --- | --- | --- | --- |
|  |  | **ICD-9** | **ICD-10** |  |
| **COMORBIDITIES** | Prior peptic ulcer or infection *H. pylori* (as a cause of ulcer) | 041.86, V12.71 | B96.81, Z87.11 | HIST_UCERA_PEP |
|  | Cancer | 140.x –165.x, 170.x – 176.x, 179, 180.x – 208.x | C00.x – C26.x, C30.x – C34.x, C37 – C39.x, C4A.x, C40.x, C41.x, C43.x – C58.x, C60.x – C86.x, C7A.x, C7B.x, C88.x, C90.x – C95.x, C96.0, C96.2x, C96.4, C96.5, C96.6, C96.9*,* Z85.x | CANCER |
|  | Thyroid disease | 240.x - 246.x | E00.x - E07.x | ENF_TIROIDEA |
|  | Diabetes Mellitus | 249.x, 250.x | E08.x – E11.x, E13.x | DM1/ DM2 |
|  | Dementia | 290.x, 291.2, 294.1x, 294.2.x | F01.x, F02.x, F03.x, F10.27, F10.97 | DEMENCIA |
|  | Sleep apnea | 327.20, 327.21, 327.23, 327.27, 327.29, 780.51, 780.53, 780.57 | G47.30, G47.31, G47.33, G47.37, G47.39 | APNEA |
|  | Hypertension | 401.x - 405.x | I10 – I13.x, I15.x, I16.x | HTA |
|  | Myocardial infarction | 410.x, 412 | I21.x, I22.x, I25.2 | IAM |
|  | Angina pectoris | 411.1, 413.x | I20.x, I25.11x, I25.7x | ANGINA |
|  | Cardiac arrhythmias (excluded AF and atrial flutter) | 427.1, 427.2, 427.4, 427.5, 427.6, 427.8, 427.9 | I47.x, I49.x | ARRITMIAS |
|  | Heart failure | 428.x, 429.4 | I50.x | ICARD |
|  | Prior stroke | V12.54, 438.x | I69.x, Z86.73 | ICTUS_PREVIO |
|  | Peripheral vascular disease | 440.x, 443.1, 443.2x, 443.8x, 443.9 | I70.x, I73.x | CLAUDICACION/ ENF_ART_PRIF |
|  | Venous thrombosis | 451.x, 453.x | I80.x, I82.x | TROMB_VENOSA |
|  | COPD | 491.2x, 492.x - 496 | J43.x, J44.x, J45.x | EPOC |
|  | Liver disease | 570, 571.3, 572.8, 573.3 | K70.4x, K71.1x, K72.x | INSUF_HEP |
|  | Chronic renal failure | 585.x | N18.x | IRC |
