# Supplementary material for: Effectiveness and safety of oral anticoagulant therapy in a real-world cohort with atrial fibrillation: The SIESTA-A study protocol
Source: PLoS One. 2023 Nov 29;18(11):e0294822. doi: 10.1371/journal.pone.0294822 (PMC10686507; doi:10.1371/journal.pone.0294822)
Supplement: S4 Table — (DOCX) [file pone.0294822.s004.docx]

**S4 Table. Concomitant medication according to Anatomical Therapeutic Chemical (ATC) codes.**

| **Medications** | **ATC-code** | **Bleeding Risk** |
| --- | --- | --- |
| Heparins and antithrombotic agents | B01AB, B01AD, B01AX | Increase |
| Antiplatelet agents | B01AC |  |
| Systemic corticosteroids | H02AA, H02AB |  |
| Non-steroidal anti-inflammatory drugs and analgesic agents | M01AB, M01AC, M01AE, M01AG, M01AH, M01AX, M01CC, M01CX, N02BA, N02BB, N02BG, N02BZ |  |
| Selective serotonin reuptake inhibitors | N06AB |  |
| Macrolide antibiotics | J01FA |  |
| H2 blockers and gastro-protective drugs | A02BA, A02BB | Decrease |
| Proton pump inhibitors | A02BC |  |
| Antihemorrhagic agents | B02AA, B02BA |  |
